# Remarks on Neuralgia

**Published:** 1825-02

**Authors:** Robert Ranking


### ART. VI.—*Remarks on Neuralgia.* By ROBERT RANKING, Esq.

AMONG the various recent and important improvements which have occurred in the department of therapeutic medicine, I may be allowed to mention the very valuable suggestions of Mr. HUTCHINSON, of Southwell, in the treatment of Neuralgia, and must solicit the indulgence of your pages for the publication of the following cases, which are, in my estimation, highly interesting.

The first I would mention is the case of a poor woman, named Woollett, residing at Mereworth, in Kent, sixty-three years of age. I was called to Mereworth Castle, to attend the Hon. Mrs. Stapleton in her accouchement, on the 19th of October, 1823. It was on the following day that Mr. Stapleton took me to see the poor woman. I found her in a most distressing state; the complaint was situated in the left side of the head and face, and the pain she appeared to be suffering was inconceivable. On inquiry, I found that she had been enduring this torturing pain for a period of sixteen weeks, without any, or scarcely any, intermission. Herself and her daughter both assured me, that she had scarcely enjoyed an hour's repose at a time, from the first commencement of the attack. In consequence of this long and continued pain, her general health had suffered a good deal; she was emaciated, her pulse quick and feeble, and her appetite much impaired. From her own account, she had taken a variety of medicines, but without experiencing any relief. At the time I saw her, she was, and had been taking for some time, the liquor arsenicalis, in doses of ten minims three times a-day; but without the least perceptible benefit. As there was no heat of skin, thirst, or fulness of pulse, to forbid the use of the ferri subcarbonas, I judged it a good case for a full and fair trial of that remedy, of late so highly and justly extolled, and recommended on a plurality of authority in every way truly respectable. My friend, Mr. Stapleton, immediately wrote to town, to Savory and Moore, for a supply; and, on the 23d of October, 1823, I commenced it in doses of two scruples ter die. I was detained at Mereworth until the 31st, at which time there was no evident amendment. I returned to Hastings on the 1st of November, and on the 4th went back to Mereworth to see Mrs. S., and was much pleased to find the poor old woman in some degree better. The paroxysms were less frequent, and upon the whole less severe. I remained at Mr. Stapleton's two days; and, Mrs. S. being quite well, I returned home, leaving an express direction that the plan the old woman was upon should be regularly persevered in, and requesting Mr. S. would give me a line in a week; which he did, conveying the pleasing intelligence that the poor woman was very much better; the paroxysms were very much diminished, both in frequency and severity; that she was able to get some refreshing sleep for three or four hours at a time: and that her appetite was improving.

Encouraged by this report, I urged the necessity of going on, begging to hear again in a fortnight; at the expiration of which time, Mr. Stapleton wrote to me, to say that the poor old creature was quite well; the pain entirely removed; that she slept the whole night; and that, to use her own expression, she was in heaven. I was at Mereworth in June last, and was much gratified to find she had passed seven months without experiencing any return. from heaven!

The next case I have the pleasure of mentioning, is that of a Mr. Welland, who was a visitor for a few weeks at Hastings. He applied to me a week before I was called to Mereworth on the above-named occasion, on account of a most severe pain in the right side of the lower jaw, extending from the angle of the jaw to the symphysis, and shooting up towards the temple. The paroxysm was immediately excited by exposure to a keen wind, but usually made its attack regularly about midnight, in so severe a way as to prevent the least sleep for three or four hours. As there existed manifest gastric derangement, I directed my remedies to that particular organ first, thinking the pain might possibly be sympathetic of such derangement; but, not finding that we gained any ground by these means, I put him upon the use of the subcarbonate of iron, in doses of half a drachm three times a-day, which, as he expressed himself, acted like a charm; for in a fortnight he was entirely relieved from the pain, which had been harrassing him for nearly three months.

Two other cases have come under my care since my successful treatment of those above related,—one in a young lady named Carruthers, the other in a female servant of a lady named Rowley, both visitors at Hastings; in both which cases, the iron proved equally successful with the two first. In all the cases, excepting the first, the astringent effects of the carbonate of iron were so great, as to require the daily use of some aperients.

I must beg leave to assert my total inability to explain the *modus operandi* of this most valuable remedial agent, in the removal of morbid derangements of the sentient organs. That it does possess a highly powerful influence over diseases of this class, the foregoing examples clearly and fully evince; and will, I trust, have the effect of confirming the testimonies of Mr. Hutchinson and of many other respectable individuals, in this particular point of practice.

*Hastings; November,* 1824.

